# Antioxidant, Anti-Inflammatory and Attenuating Intracellular Reactive Oxygen Species Activities of *Nicotiana tabacum* var. Virginia Leaf Extract Phytosomes and Shape Memory Gel Formulation

**DOI:** 10.3390/gels9020078

**Published:** 2023-01-18

**Authors:** Chuda Chittasupho, Kunyakorn Chaobankrang, Araya Sarawungkad, Weerasak Samee, Sudarshan Singh, Kirachuda Hemsuwimon, Siriporn Okonogi, Kantaporn Kheawfu, Kanokwan Kiattisin, Wantida Chaiyana

**Affiliations:** 1 Department of Pharmaceutical Sciences, Faculty of Pharmacy, Chiang Mai University, Mueang, Chiang Mai 50200, Thailand; 2 Research Center of Pharmaceutical Nanotechnology, Faculty of Pharmacy, Chiang Mai University, Chiang Mai 50200, Thailand; 3 Department of Pharmaceutical Chemistry, Faculty of Pharmacy, Srinakharinwirot University, Ongkharak, Nahonnayok 26120, Thailand; 4 Tobacco Authority of Thailand, Leaf Department, Maejo Tobacco Experiment Station, Chiang Mai 50290, Thailand

**Keywords:** phytosomes, tobacco leaf, polyurethane-62, reactive oxygen species, shape memory gel

## Abstract

Oxidative stress is one of the major causes of skin aging. In this study, the shape memory gels containing phytosomes were developed as a delivery system for *Nicotiana tabacum* var. Virginia fresh (VFL) and dry (VDL) leaf extracts. The extracts were loaded in the phytosomes by a solvent displacement method. The physical and chemical characteristics and stability of phytosomes were evaluated by dynamic light scattering and phytochemistry, respectively. The in vitro antioxidant activity and intracellular reactive oxygen species reduction of phytosomes and/or extracts were investigated by the DPPH and ABTS radical scavenging assays, FRAP assay, and DCFH-DA fluorescent probe. The cytotoxicity and anti-inflammatory activity of VDL and VFL phytosomes were studied by an MTT and a nitric oxide assay, respectively. Here, we first reported the total phenolic content in the dry leaf extract of *N. tabacum* var. Virginia was significantly greater than that of the fresh leaf extract. The HPLC analysis results revealed that VDL and VFL extracts contained 4.94 ± 0.04 and 3.13 ± 0.01 µg/mL of chlorogenic acid and 0.89 ± 0.00 and 0.24 ± 0.00 µg/mL of rutin, respectively. The phytosomes of the VDL and VFL extracts displayed stable size, polydispersity index, zeta potential values, and good chemical stability. VDL and VDL phytosomes showed higher phenolic and flavonoid contents which showed stronger DPPH and ABTS radical scavenging effects and reduced the intracellular ROS. The results suggested that the phenolic compounds are the main factor in their antioxidant activity. Both VDL and VFL phytosomes inhibited nitric oxide production induced by LPS, suggesting the anti-inflammatory activity of the phytosomes. The shape memory gel containing VDL and VFL phytosomes had good physical stability in terms of pH and viscosity. The VDL and VFL phytosomes dispersed in the shape memory gels can be considered as a promising therapeutic delivery system for protecting the skin from oxidation and reactive oxygen species.

## 1. Introduction

Skin aging can be caused by internal and external factors [1]. The internal factors include chronological aging and hormonal deficiency, resulting in the deterioration of tissues in the dermis and epidermis. The decrease in the number of fibroblasts synthesizing collagen, elastin fiber, and glycosaminoglycan leads to an increase in laxity and wrinkles [2]. The external factors are ultraviolet radiation (UVR), nutrition, smoking, and air pollution, causing increased free radicals and oxidative stress in the skin [3]. Oxidative stress can cause skin wrinkling and is linked to human skin diseases, including skin cancer. Reactive oxygen species (ROS) are involved in the pathogenesis of several allergic and inflammatory skin diseases. Intracellular and extracellular oxidative stress-initiated ROS promote skin aging. ROS disrupt gene and protein function, change intracellular and extracellular homeostasis, and impair skin function [4]. The prolonged accumulation of ROS can result in cellular aging and may adversely affect health. These reactions involve the damage of lipids, proteins, and DNA, thus, causing cellular damage that can eventually lead to cell death [5].

Antioxidants resist or slow down the aging of the skin. Several formulations have been developed to deliver antioxidants to prevent or delay the deterioration of skin cells. Antioxidants in nature are usually phenolic compounds, flavonoids, and tannins. In this study, the biological activity, and phytochemical constituents of tobacco dry leaf extract (VDL) and fresh leaf extract (VFL) from *Nicotiana tabacum* var. Virginia, especially phenolic compounds and flavonoids, were investigated. Although these phytochemicals have good antioxidative activity due to their ability to bind free radicals with the hydroxyl functional group (-OH) in the chemical structure, most effective natural extracts have limited water solubility [6,7,8,9,10,11]. Therefore, the delivery system must be developed to increase the solubility and stability of the bioactive compounds in the extracts.

The phytosome is a type of drug delivery system. The phytosome encapsulates natural bioactive constituents by forming hydrogen bonds with the polar head of the phytosome. The phytosome and bioactive compound complexes are formed in which the phospholipids’ head group is anchored. In contrast, the fatty acid chains of phospholipids encapsulate the polar part of complexes to form a lipophilic surface [12]. Phytosomes can be prepared by various methods, including solvent evaporation, co-solvent lyophilization, and antisolvent precipitation [13]. In this study, the solvent displacement method was applied to fabricate phytosomes. Generally, a polar aprotic solvent was used to dissolve the drug and phytosome component to support an optimal bonding environment. The extract was encapsulated in the phytosomes by forming hydrogen bonds with phospholipids to improve drug retention, enhance the stability of the formulation, increase the permeability and drug absorption through the skin, and strengthen the bioactive compound efficiency [14,15,16]. Numerous studies have shown the success of developing phytosome formulations for the delivery of natural substances such as apigenin, *Centella asiatica* extract, and grape seed (*Vitis vinifera* L.) extract [15,16,17]. Phytosomes stabilized and increased the bioavailability and permeability of the bioactive compounds.

Natural antioxidants, whether in the form of raw extracts or chemical constituents, are extremely effective at preventing the damaging processes caused by oxidative stress [18]. Despite the fact that the toxicity profile of most medicinal plants has not been thoroughly evaluated, it is widely accepted that medicines derived from a plant extract are safer than their synthetic counterparts [19,20]. The search for novel natural antioxidants of plant origin has intensified. The antioxidant activity of plant extract plays a significant role in protecting against the non-communicable diseases caused by oxidative stress. The current study was designed to investigate the possibility of *N. tabacum* var. Virginia dry and fresh leaf extracts for preventing skin aging caused by oxidative stress. Therefore, the total phenolic content and total flavonoids and the antioxidant activities of ethanol extract of *N. tabacum* var. Virginia dry and fresh leaf were investigated. The VDL and VFL phytosomes were successfully formulated with good colloidal stability. We found that the storage temperature and phytosome formulation including phospholipids, cholesterol, and poloxamers played significant roles in the stability of the VDL and VFL phytosomes. Pearson correlation coefficients revealed the very high correlation between the total phenolic content and antioxidant activity of the extract and phytosomes. The effective and non-toxic concentrations of VDL and VFL phytosomes in reducing inflammation and intracellular reactive oxygen species in keratinocytes were reported. The gels containing phytosomes were developed to make phytosomes feasible for topical application. The method to prepare the shape memory gel containing phytosome developed in this study was simple and was achievable for scaling up commercially.

## 2. Results and Discussion

### 2.1. Physical Characterization and Colloidal Stability of VDL and VFL Phytosomes, Phytosomes w/o Chloresterol, Phytosomes w/o Poloxamer, and Nanoparticles 

**Figure 1:**
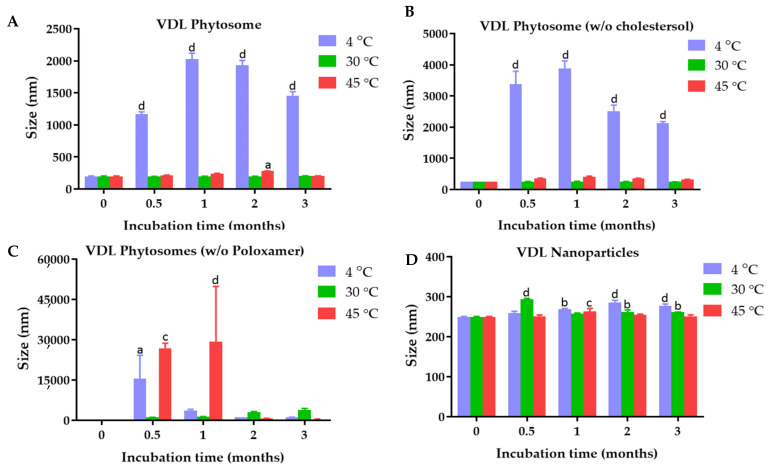
Size of (**A**) VDL phytosomes, (**B**) VDL phytosomes (w/o cholesterol), (**C**) VDL phytosomes (*w*/*o* poloxamer), and (**D**) VDL nanoparticles after fresh preparation and storage for 0.5, 1, 2, and 3 months at 4 °C, 30 °C, and 45 °C. The letters a, b, c, and d indicate *p*-values < 0.05, 0.01, 0.001, and 0.001, respectively.

**Figure 2:**
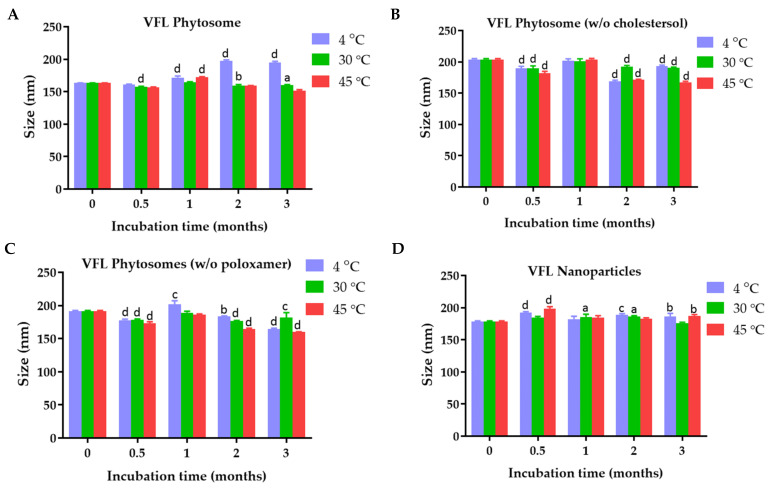
Size of (**A**) VFL phytosomes, (**B**) VFL phytosomes (*w*/*o* cholesterol), (**C**) VFL phytosomes (*w*/*o* poloxamer), and (**D**) VFL nanoparticles after fresh preparation and storage for 0.5, 1, 2, and 3 months at 4 °C, 30 °C, and 45 °C. The letters a, b, c, and d indicate *p*-value < 0.05, 0.01, 0.001, and 0.001, respectively.

The average particle size and stability of the VDL and VFL phytosomes with and without cholesterol or poloxamer, and VDL and VFL nanoparticles are shown in Figure 1. The size of the VDL phytosome with cholesterol, VDL phytosome without cholesterol, VDL phytosome without poloxamer, and VDL nanoparticles were 198.17 ± 6.63 nm, 248.63 ± 1.33 nm, 311.00 ± 5.21 nm, and 249.30 ± 1.56 nm, respectively. The size of the VFL phytosome with cholesterol, VFL phytosome without cholesterol, VFL phytosome without poloxamer, and VFL nanoparticles shown in Figure 2 were 163.40 ± 0.26 nm, 203.83 ± 1.44 nm, 191.43.00 ± 1.19 nm, and 178.67 ± 0.82 nm, respectively.

The results showed that the particle size significantly increased in the absence of cholesterol in both VDL and VFL phytosome formulations. The importance of cholesterol in liposomes was reported. The solidity of the liposomes depended on the content of cholesterol. Numerous studies used cholesterol as a stabilizer of liposomes because it helped the packing of phospholipid molecules, reduced bilayer permeability, prevented liposome aggregation, and increased the rigidity and resistance to shear stress of the lipid bilayer [21]. Here, 0.9% *w*/*w* of cholesterol was used and was considered optimal for VDL and VFL phytosome formulations. In the absence of 0.1% *w*/*v* poloxamer 407, the VDL and VFL phytosomes were also significantly larger than the phytosomes stabilized with poloxamer. Poloxamer was assumed to be attached to the phytosome by adsorption. Minnelli et al. showed that combining poloxamer 407 with liposome increased liposome stability by hindering liposome aggregation, shielding the colloidal surface with the hydrophilic portions of the polymer, and decreasing the fusion of phosphatidylcholine multilamellar vesicles [22]. The size of the phytosomes significantly increased when samples were stored at 4 °C. Generally, the size of the liposome/phytosome is influenced by the elasticity modulus of the lipid bilayer, which depends on the temperature below the phase transition. Most membrane compositions form larger liposomes/phytosomes close to or below the gel-to-liquid crystalline phase transition temperature, where the membrane elasticity modulus is much larger [23]. The colloidal stability study results suggested that VDL and VFL phytosomes and nanoparticles should be stored at 30 °C to maintain the size of the particles. Interestingly, the effects of membrane stiffness of phytosomes were not found to significantly affect the size of VFL phytosomes when stored at 4 °C. This result suggested that the stability of phytosomes was mostly dependent on the zeta potential values of the phytosomes, which defeated the effect of the membrane elasticity [24].

**Figure 3:**
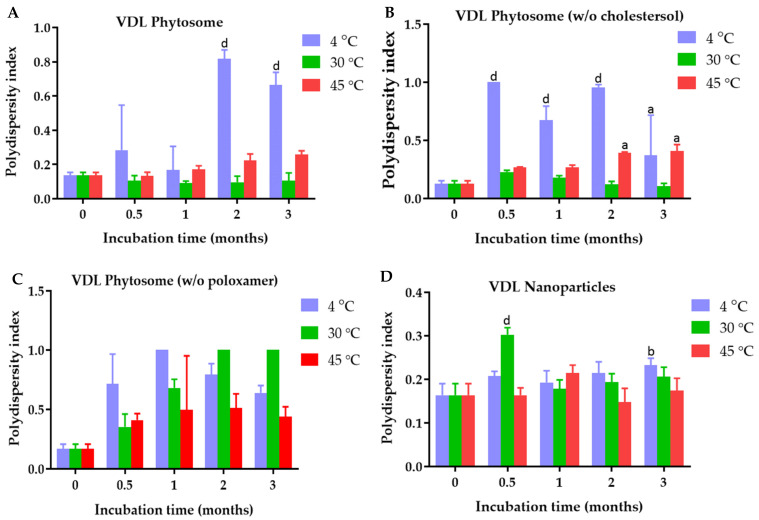
Polydispersity index of (**A**) VDL phytosomes, (**B**) VDL phytosomes (*w*/*o* cholesterol), (**C**) VDL phytosomes (*w*/*o* poloxamer), and (**D**) VDL after fresh preparation and storage for 0.5, 1, 2, and 3 months at 4 °C, 30 °C, and 45 °C. The letters a, b, and d indicate *p*-values < 0.05, 0.01, and 0.001, respectively.

**Figure 4:**
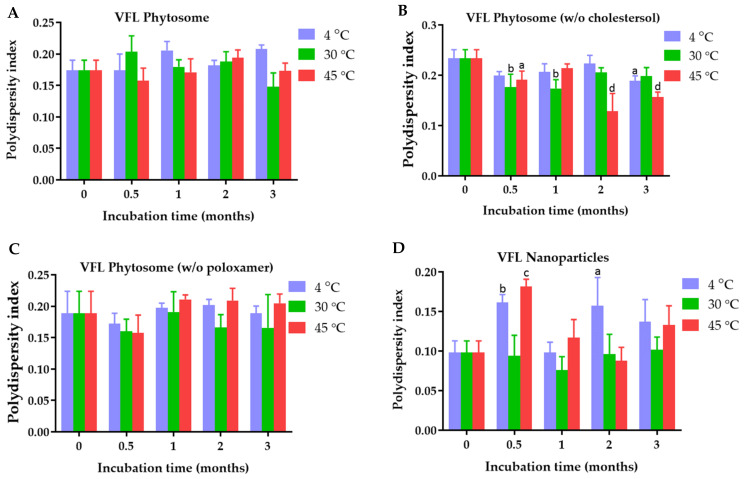
Polydispersity index of (**A**) VFL phytosomes, (**B**) VFL phytosomes (*w*/*o* cholesterol), (**C**) VFL phytosomes (*w*/*o* poloxamer), and (**D**) VFL nanoparticles after fresh preparation and storage for 0.5, 1, 2, and 3 months at 4 °C, 30 °C, and 45 °C. The letters a, b, c, and d indicate *p*-values < 0.05, 0.01, 0.001, and 0.001, respectively.

The polydispersity index of VDL phytosome with cholesterol, VDL phytosome without cholesterol, VDL phytosome without poloxamer, and VDL nanoparticles were 0.136 ± 0.019, 0.128 ± 0.024, 0.169 ± 0.040, and 0.164 ± 0.027, respectively (Figure 3). The polydispersity index of VFL phytosome with cholesterol, VFL phytosome without cholesterol, VFL phytosome without poloxamer, and VFL nanoparticles were 0.174 ± 0.016, 0.234 ± 0.017, 0.189 ± 0.035, and 0.098 ± 0.015, respectively (Figure 4).

The phytosome and nanoparticle size distribution can be articulated through the polydispersity index value. The nanoparticles with PDI values less than 0.3 are considered monodisperse, while a PDI value higher than 0.7 indicates a polydisperse system [25]. All VDL and VFL phytosomes and nanoparticles had PDI values less than 0.2, suggesting the uniformity of a sample based on the size. Heterogenicity can occur due to phytosome or nanoparticle aggregation. The polydispersity index of VDL phytosomes without poloxamer significantly increased to be higher than 0.4 when the phytosomes were stored at 4 °C, 30 °C, and 45 °C. VDL phytosomes containing poloxamers with and without cholesterol showed higher PDI values when they were kept at 4 °C. These results might be due to the aggregation of phytosomes at low temperatures. The poloxamer increased colloidal stability by forming hydrophilic layers of polyoxyethylene oxide at the surface of phytosomes and nanoparticles, hence preventing particle aggregation attributed to van der Waals forces between phytosomes and nanoparticles [26].

**Figure 5:**
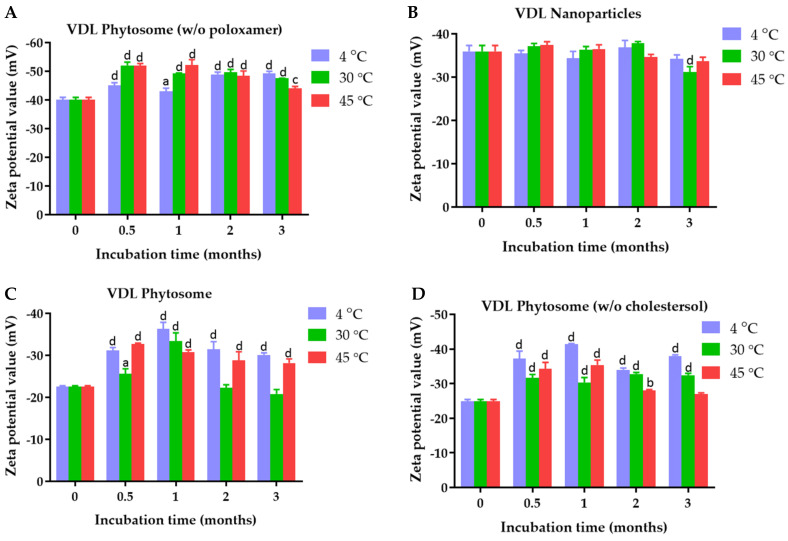
Zeta potential values of (**A**) VDL phytosomes, (**B**) VDL phytosomes (*w*/*o* cholesterol), (**C**) VDL phytosomes (*w*/*o* poloxamer), and (**D**) VDL nanoparticles after fresh preparation and storage for 0.5, 1, 2, and 3 months at 4 °C, 30 °C, and 45 °C. The letters a, b, c, and d indicate *p*-values < 0.05, 0.01, 0.001, and 0.001, respectively.

**Figure 6:**
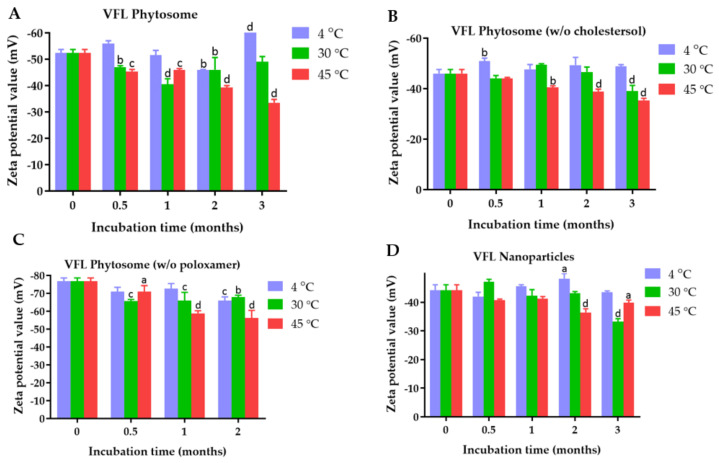
Zeta potential values of (**A**) VFL phytosomes, (**B**) VFL phytosomes (*w*/*o* cholesterol), (**C**) VFL phytosomes (*w*/*o* poloxamer), and (**D**) VFL nanoparticles after fresh preparation and storage for 0.5, 1, 2, and 3 months at 4 °C, 30 °C, and 45 °C. The letters a, b, c, and d indicate *p*-values < 0.05, 0.01, 0.001, and 0.001, respectively.

The zeta potential values of VDL phytosome with cholesterol, VDL phytosome without cholesterol, VDL phytosome without poloxamer, and VDL nanoparticles were −22.57 ± 0.23 mV, −24.93 ± 0.49 mV, −40.07 ± 0.86 mV, and −35.97 ± 01.36 nm, respectively (Figure 5). The zeta potential values of VFL phytosome with cholesterol, VFL phytosome without cholesterol, VFL phytosome without poloxamer, and VFL nanoparticles were −52.33 ± 1.40 mV, −46 ± 1.65 mV, −76.93 ± 1.71 mV, and −44.23 ± 1.86 mV, respectively (Figure 6).

Figure 5 and Figure 6 show the results of the zeta potential measurements of VDL and VFL phytosomes and nanoparticles as a function of the storage time and temperature. The zeta potential value higher than ±30 mV suggested high colloidal stability of the phytosomes and nanoparticles.

Zeta potential results showed that all VDL and VFL formulations exhibited a negative charge with values ranging from 22.57 ± 0.23 mV to −40.07 ± 0.86 mV, and −44.23 ± 01.86 mV to 76.93 ± 1.71 mV, respectively. The negative charge value was due to the presence of phosphate and carbonyl groups of phosphatidylcholines. Compared with phytosomes coated with poloxamer, a more negative charge of phytosomes and nanoparticles was observed with phytosomes prepared without poloxamer. This could be because poloxamer adsorption on the phytosome or nanoparticle surface forms a coating layer, shielding the negative surface charge and shifting the plane of shear away from the particle surface. Overall, phytosomes containing cholesterol and poloxamer encapsulating VDL and VFL were the best formulation with optimal size, PDI, and zeta potential values. Both formulations were recommended to store at 30 °C.

Phytosomes of herbal extracts have been developed and characterized. Tiwari et al. developed herbal extract-loaded phytosomes. They showed that the encapsulation of herbal extract in the phytosomes did not change the chemical structure due to an FTIR analysis [27]. Direito et al. reported that the size of phytosomes depends on the amount of lipid composition in the formulation. It has been reported that increasing phospholipids in the phytosome increased the tendency of agglomeration [28]. The surface charge expressed as the zeta potential is an important physicochemical parameter that influences the stability of nanosuspensions which may also influence the biodistribution, pharmacokinetics, cellular affinity, and drug internalization [29]. When compared to a positive surface charge, the negative zeta potential is generally associated with higher biocompatibility [30,31].

### 2.2. Total Phenolic and Flavonoid Contents of VDL and VFL Phytosomes, Phytosomes w/o Cholesterol, Phytosomes w/o Poloxamer, and Nanoparticles

**Figure 7:**
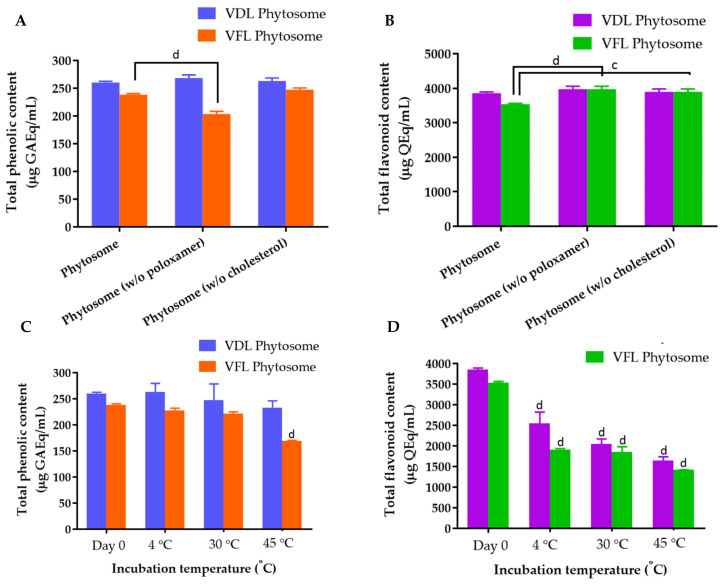
(**A**) Total phenolic content in various concentrations of VDL and VFL phytosomes. (**B**) Total flavonoid content in various concentrations of VDL and VFL phytosomes. (**C**) Total phenolic content and (**D**) total flavonoid content in VDL and VFL phytosomes upon storage at 4, 30, and 45 °C for 3 months. The letters a, b, c, and d indicate *p*-values < 0.05, 0.01, 0.001, and 0.001, respectively.

The total phenolic content in the VDL and VFL ethanolic extracts using the Folin–Ciocalteu reagent was expressed in terms of the gallic acid equivalent. The standard curves plotted between the absorbance, and gallic acid reacted with the Folin–Ciocalteau reagent are shown in Figure S1A. The total phenolic contents in the VDL and VFL extracts were 265 ± 0.67 and 258.56 ± 0.81 mg gallic acid equivalent/mL crude extract, respectively. The phytosomes of VDL and VFL contained 260.35 ± 2.29 and 238.54 ± 1.86 mg gallic acid equivalent/mL (Figure 7A). Here, we first reported the total phenolic content in the dry leaf extract of *N. tabacum* var. Virginia was significantly greater than that of the fresh leaf extract.

The standard curves of quercetin and EGCG reacted with the aluminum chloride reagent were presented in Figures S1B and 1C, respectively. The total flavonoid contents in the VDL and VFL extracts were 3620.52 ± 106.01 mg and 2836.44 ± 232.99 mg quercetin equivalent/mL crude extract, respectively. VDL and VFL extracts contained 3966.09 ± 388.49 mg and 2656.71 ± 247.24 mg EGCG equivalent/mL crude extract, respectively. The phytosomes of VDL and VFL contained 3858.19 ± 33.64 mg and 3538.41 ± 27.29 mg quercetin equivalent/mL phytosome, and 4343.46 ± 37.84 mg and 3938.71 ± 30.70 mg EGCG equivalent/mL phytosome, respectively (Figure 7B). The results suggested that the VDL extract and phytosome contained a larger amount of flavonoids compared with the VFL extract and phytosome. The component of VDL phytosomes did not affect the encapsulation of the total phenolic and total flavonoids, but the encapsulation of the total phenolic and total flavonoids of VFL was affected by the VFL phytosome formulation. The stability of the total phenolic and total flavonoids in VDL and VFL phytosomes was temperature dependent. The total phenolic and flavonoid contents significantly reduced when the phytosomes were stored at 45 °C (Figure 7C). The total flavonoids were prone to degrade more easily compared with total phenolic compounds encapsulated in the phytosomes (Figure 7D).

### 2.3. Antioxidant Activities of VDL and VFL Extracts and Phytosomes

**Figure 8:**
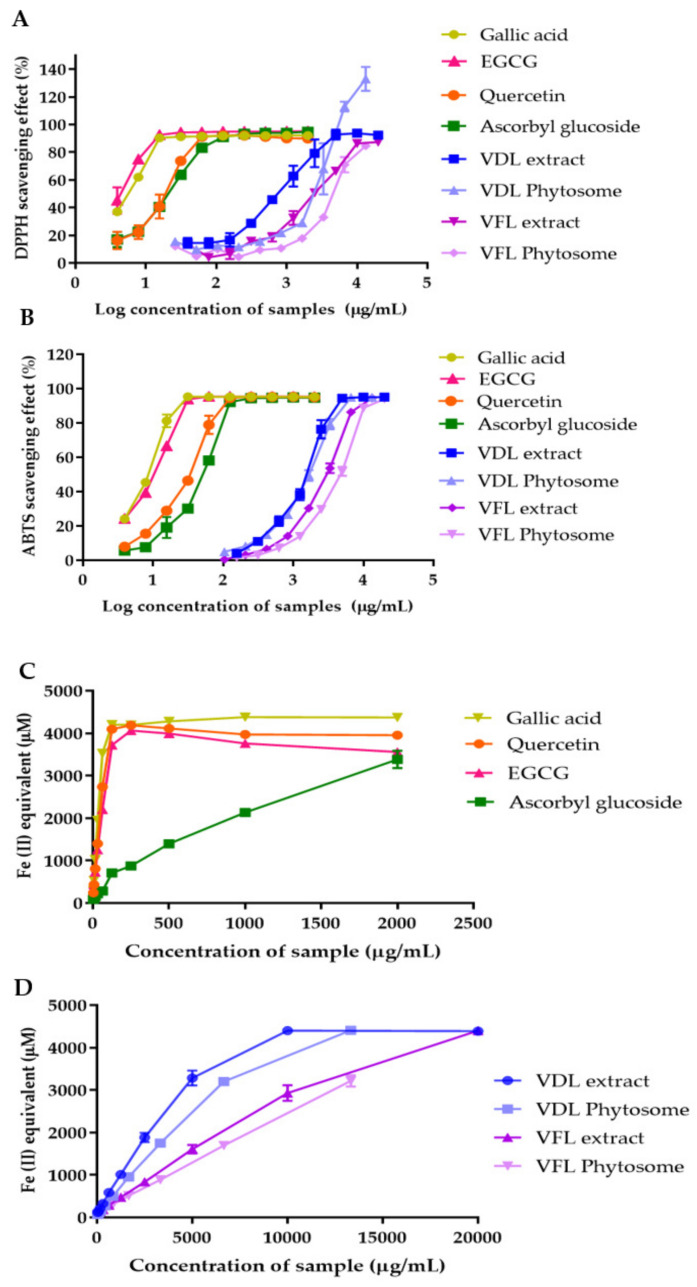
The DPPH scavenging activity of gallic acid, quercetin, epigallocatechin, ascorbyl glucoside, VDL and VFL extract and phytosomes determined by (**A**) DPPH free radical scavenging assay; (**B**) ABTS free radical scavenging assay; (**C**) ferric reducing antioxidant power assay for gallic acid, quercetin, and ascorbyl glucoside; (**D**) ferric reducing antioxidant power assay for VDL and VFL extracts and phytosomes.

The antioxidant activities of VDL and VFL phytosomes were determined by DPPH and ABTS radical scavenging assays. The scavenging activity of the extract and phytosomes was compared to that of gallic acid, quercetin, ascorbyl glucoside, and EGCG. The IC_50_ values obtained from the DPPH method were 5.40 µg/mL, 17.39 µg/mL, 19.74 µg/mL, and 4.82 µg/mL for gallic acid, quercetin, ascorbyl glucoside, and EGCG, respectively (Figure 8A). The IC_50_ values for VDL extract, VFL extract, VDL phytosomes, and VFL phytosomes were 693.70 µg/mL, 3363 µg/mL, 2146 µg/mL, and 4377 µg/mL, respectively (Figure 8A). The IC_50_ values obtained from the ABTS radical scavenging capacity of gallic acid, quercetin, ascorbyl glucoside, EGCG, VDL extract, VFL extract, VDL phytosomes, and VFL phytosomes were 7.87 µg/mL, 29.11 µg/mL, 46.3 µg/mL, 9.39 µg/mL, 1400 µg/mL, 2733 µg/mL, 1528 µg/mL, and 4154 µg/mL, respectively (Figure 8B).

The FRAP method measured the antioxidant and reduction ability of samples according to Fe^3+^ to Fe^2+^ reducing activity. Gallic acid, quercetin, ascorbyl glucoside, and EGCG at 250 µg/mL reduced Fe^3+^ to 4206.15 µM, 4097.15 µM, 711.72 µM, and 3721.07 µM of Fe^2+^, respectively (Figure 8C). The VDL extract, VDL phytosomes, VFL extract, and VFL phytosomes at 250 µg/mL exhibited 306.61 µM, 195.36 µM, 169.76 µM, and 139.80 µM, respectively, suggesting the higher reducing power capacity of VDL compared with VFL (Figure 8D).

The results revealed that both the VDL extract and phytosomes had higher DPPH and ABTS radical scavenging activity and ferric reducing power than the VFL extract and phytosomes. Our results indicated that the VDL extract and VDL phytosomes demonstrated stronger DPPH and ABTS radical scavenging effects, probably because they contained higher amounts of phenolic and flavonoid contents, compared with VFL and VFL phytosomes. These results suggested that phenolic compounds are the main factor in their antioxidant activity.

### 2.4. Correlation between the Total Phenolic and Total Flavonoid Content and Antioxidant Activities

The correlation between the phenolic and flavonoid contents and antioxidant activities was evaluated by the Pearson’s correlation test shown in Table 1. The total phenolic and total flavonoids in the VDL phytosomes, VFL extract, and VFL phytosomes showed a very high correlation with all three antioxidant activities. The total flavonoids in the VDL extract showed a high correlation with the DPPH free radical scavenging activity. These results supported the fact that the phenolic and flavonoids in the VDL and VFL extracts and phytosomes could scavenge free radicals and reduce ferric ions.

The total phenolic content and total flavonoid contents reported by Yati et al. were lower than our findings [32]. This might be due to the fact that the solvent used for polyphenol extraction is different. The higher effectiveness of ethanol in extracting phenolic compounds and flavonoids was shown in previous studies [33,34,35,36]. The ethanol extracts also exhibited a higher activity than the aqueous extract [33,34,35]. The extracts containing more polyphenols had higher antioxidant activity. Therefore, the 95% ethanol solvent might be more suitable to extract phenolic and flavonoids from *Nicotiana tabacum* var. Virginia.

### 2.5. HPLC Analysis of VDL and VFL Extracts

**Figure 9:**
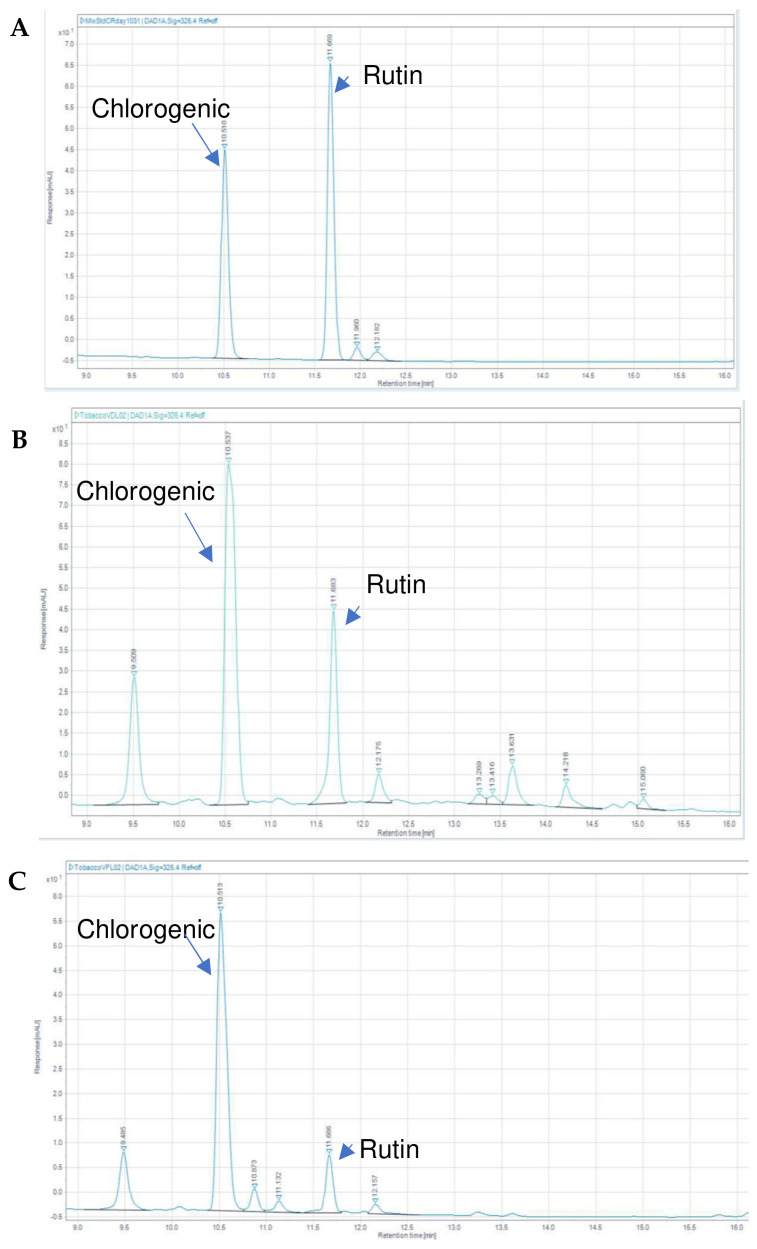
HPLC chromatograms of (**A**) chlorogenic acid and rutin standards at a retention time of 10.51 min and 11.68 min, respectively. (**B**) VDL extract and (**C**) VFL extract.

Chlorogenic acid and rutin have been reported as the main phenolic compounds in tobacco leaves. Chen et al. have shown that chlorogenic acid and rutin concentration were the highest among the other polyphenol compounds in tobacco leaves [37]. A reverse-phased HPLC was applied to analyze polyphenols in the VDL and VFL extracts. The HPLC profiles of the standard chlorogenic acid and rutin are presented in Figure 9. Chlorogenic acid and rutin were detected with high concentrations in the VDL and VFL extracts. The VDL and VFL extracts contained 4.94 ± 0.04 and 3.13 ± 0.01 µg/mL of chlorogenic acid, respectively, and 0.89 ± 0.00 and 0.24 ± 0.00 µg/mL of rutin, respectively. According to the literature, the peak at a retention time of 9.5 min might be assigned for nicotine [38]. At a retention time of 10.87 min and 11.13 min, the peaks might be identified as isomers of chlorogenic acid (3-caffoylquinic acid), such as 4-caffoylquinic acid and 5-caffoylquinic acid, respectively [39].

### 2.6. Effects of VDL and VFL Extracts and Phytosomes on Keratinocyte Cell Viability

**Figure 10:**
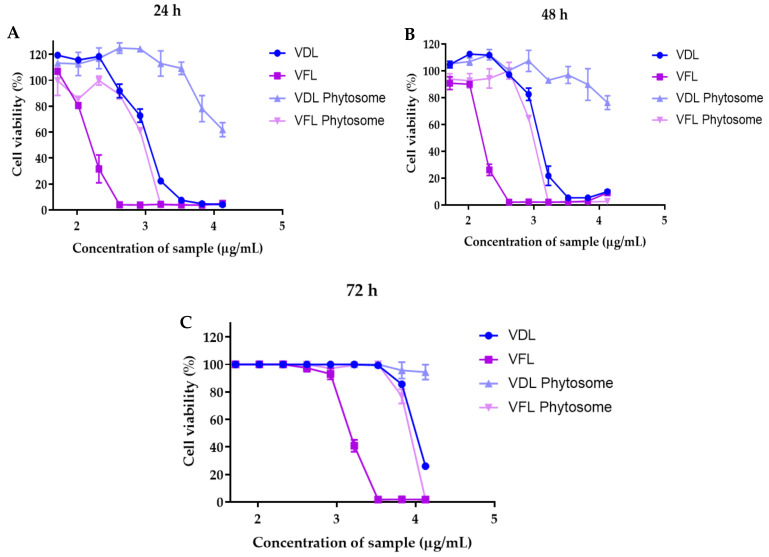
The viability and growth of HaCaT cells treated with various concentrations of the VDL and VFL extracts and phytosomes for (**A**) 24 h, (**B**) 48 h, and (**C**) 72 h.

The IC_50_ values of the VDL extract, VFL extract, VDL phytosomes, and VFL phytosomes after exposure to HaCaT cells for 24 h were 1137 µg/mL, 164.8 µg/mL, 15,741 µg/mL, and 910.5 µg/mL, respectively (Figure 10A). The IC_50_ values of the VDL extract, VFL extract, VDL phytosomes, and VFL phytosomes increased upon exposure with HaCaT cells for 48 h to 1222 µg/mL, 166 µg/mL, 28,671 µg/mL, and 916.4 µg/mL, respectively (Figure 10B). After 72 h, the IC_50_ values of the VDL extract, VFL extract, VDL phytosomes, and VFL phytosomes were 10,326 µg/mL, 1528 µg/mL, 139,737 µg/mL, and 7805 µg/mL, respectively (Figure 10C). The IC_50_ values of the VFL extract and VFL phytosomes were less than that of the VDL extract and VDL phytosomes, indicating the higher cytotoxicity against human keratinocyte cells. The IC_50_ values of all extracts and phytosomes increased with the incubation time, suggesting that VDL and VFL extracts and their phytosomes were safe to apply on skin. Phytosomes had higher IC_50_ values compared to the extract, indicating that they can protect cells from cytotoxic agents in the extracts.

### 2.7. Reactive Oxygen Species’ Levels in HaCaT Cells Exposed to VDL and VFL Phytosomes

**Figure 11:**
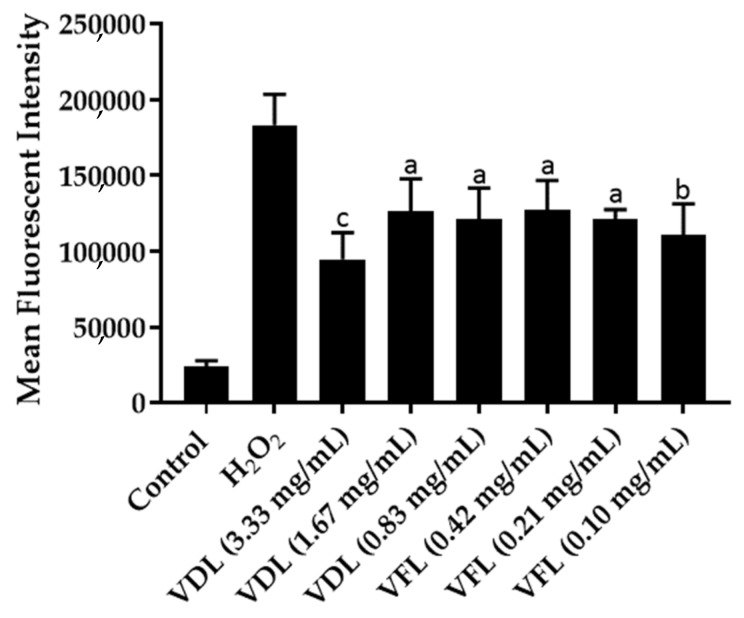
Intracellular ROS levels assayed with DCFH-DA fluorescent probe HaCaT cells treated with VDL and VFL phytosomes. The letters a, b, and c indicate *p*-values < 0.05, 0.01, and 0.001, respectively.

Intracellular ROS were assayed using fluorescent probe DCFH-DA, which can cross cell membranes and oxidize to a fluorescent DCF by intracellular ROS. Compared with the hydrogen peroxide-induced HaCaT cells, the fluorescent intensity of cells treated with VDL and VFL phytosomes was significantly decreased, indicating that the intracellular ROS level was significantly decreased (Figure 11). The results suggested that VDL and VFL phytosomes reduced the intracellular ROS, probably due to the total phenolic and total flavonoid compounds acting as an antioxidant in the extract.

Free radicals and other ROS such as the oxygen singlet free radical, hydroxyl radical, peroxyl radical, and nitric oxide free radical are continuously formed at low concentrations during normal essential metabolic processes [40]. The natural antioxidant system regulates the amount of these free radical species to maintain redox hemostasis [41]. Polyphenols are strong antioxidants that can neutralize free radicals by donating an electron or hydrogen atom to a free radical through the H-atom transfer mechanism [42]. Tobacco leaves contain significant concentrations of polyphenols and carotenoids, which are important naturally occurring antioxidants [43,44]. Although we are the first to show the intracellular ROS suppression of *N. tabacum* leaves, the antioxidant activities of *N. tabacum* leaves were expressed in several studies by scavenging activities on hydroxyl, superoxide anion, DPPH and ABTS radicals, ferric thiocyanate forming complex, and reducing power [32,45,46]. These results supported the intracellular ROS inhibition of *N. tabacum* leaves firstly presented in our study.

### 2.8. Inhibition of LPS-Induced Nitric Oxide Production

**Figure 12:**
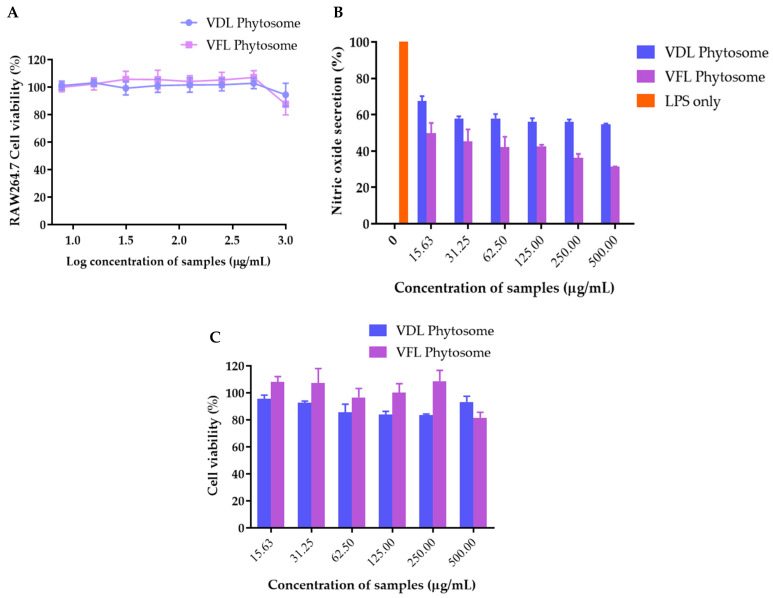
(**A**) RAW264.7 cell viability after the treatment with VDL and VFL phytosomes. (**B**) Effect of VDL and VFL phytosomes on NO production in LPS-stimulated RAW 264.7 cells. (**C**) RAW264.7 cell viability after the nitric oxide inhibition experiment.

The effects of VDL and VFL phytosomes on RAW264.7 cell viability are shown in Figure 12A. The viability of RAW264.7 cells treated with 7.8–500 µg/mL was not significantly reduced, indicating that phytosomes at this concentration range showed no cytotoxic effect on RAW264.7 cells. Natural polyphenols have demonstrated anti-inflammatory activity in vitro and in vivo, highlighting their therapeutic applications in a variety of diseases. Numerous studies have shown the anti-inflammatory and immune modulation activities of polyphenols [15,21]. The ability of these natural compounds to modulate the expression of several pro-inflammatory genes such as multiple cytokines, lipoxygenase, nitric oxide synthases, and cyclooxygenase, in addition to their antioxidant properties such as ROS scavenging, helps to regulate inflammatory signaling [22,23]. Thus, VDL and VFL phytosomes at 15.6–500 µg/mL were further used to study the anti-inflammatory effect of the phytosomes by inhibiting nitric oxide production. RAW264.7 cells were stimulated with LPS with or without co-treatment with VDL and VFL phytosomes. As shown in Figure 12B, both VDL and VFL phytosomes inhibited the NO production induced by LPS. The VFL phytosome inhibited LPS-induced NO production in RAW264.7 cells to a greater extent than the VDL phytosome. The viability of RAW264.7 cells treated with LPS and samples were not changed during the experiment (Figure 12C). A previous study on the phytoconstituent isolated from *N. tabacum* demonstrated significant inhibition of COX-2 by the downregulation of COX-2 mRNA [47]. Therefore, our data suggested that phytosomes that incorporated *N. tabacum* Virginia leaf extract can be considered as a potential candidate for the mitigation of dermal inflammations.

### 2.9. Characterization of Shape Memory Gel Containing VDL and VFL Phytosomes

**Figure 13:**
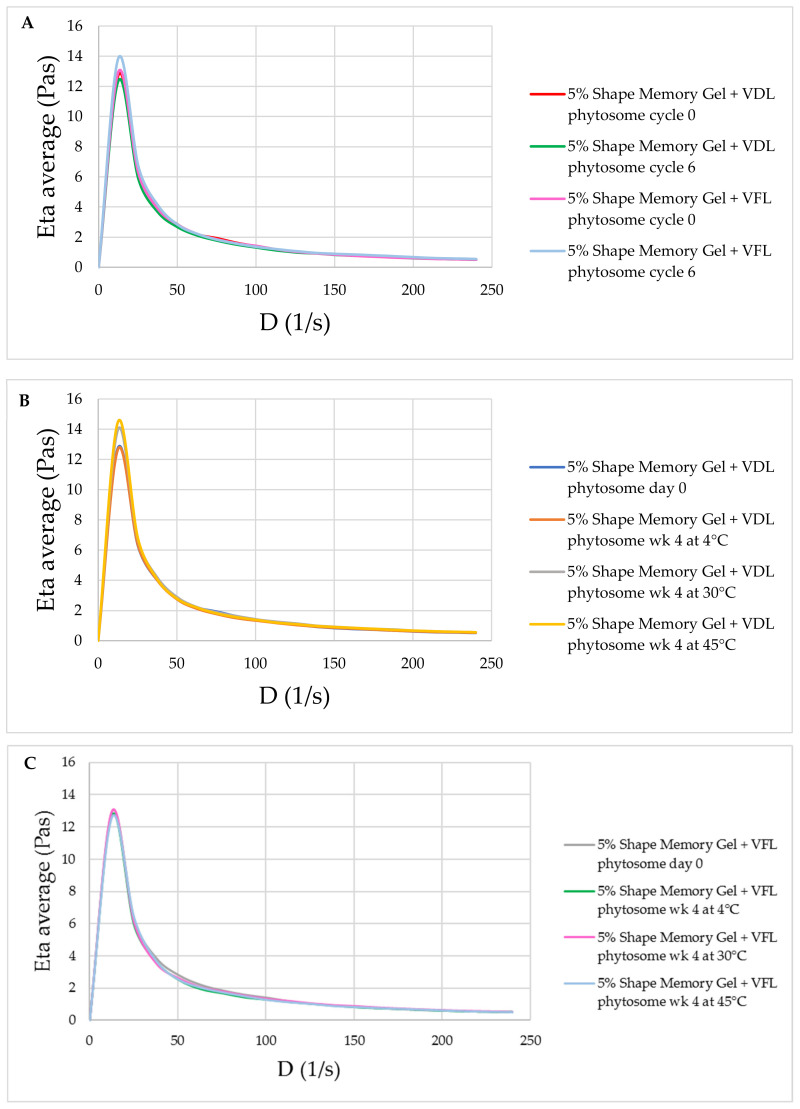
Rheology profile of the gels. (**A**) Flow curves of gels containing 1% *w*/*w* VDL and VFL phytosomes after six heating–cooling cycles of stability study. Flow curves of gels containing 1% *w*/*w* (**B**) VDL and (**C**) VFL phytosomes after 1 month-storage at 4 °C, 30 °C, and 45 °C, expressed as viscosity and shear rate.

**Figure 14:**
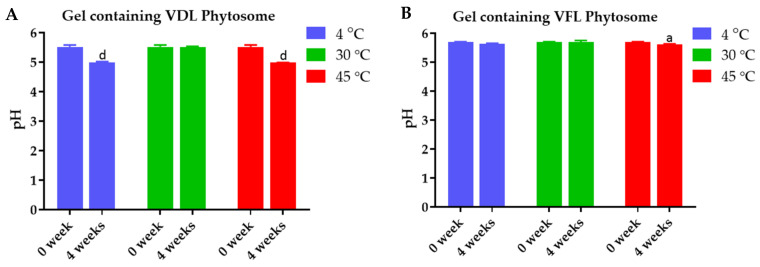
pH the gels. (**A**) pH of gels containing 1% *w*/*w* VDL phytosome after 1 month-storage at 4 °C, 30 °C, and 45 °C of stability study. (**B**) pH of gels containing 1% *w*/*w* VFL phytosome after 1 month-storage at 4 °C, 30 °C, and 45 °C of stability study. The letters a and d indicate *p*-values < 0.05 and 0.01, and 0.001, respectively.

The physical stability of the shape memory gel containing VDL and VFL phytosomes was confirmed by the maintained appearance, viscosity, and pH. The appearance of gels is shown in Figure S2. Figure 13 exhibits the change in viscosity for gels containing 0.1% *w*/*w* VDL and VFL phytosomes as a function of shear rate. All samples showed a similar trend of change in viscosity. Initially, the gel showed high viscosity, followed by a gradual decrease in viscosity as the shear rate was applied, and followed by a plateau region. These results suggested that the gel exhibited pseudoplastic rheology behavior. The viscosity of gels containing VDL and VFL phytosomes was not significantly changed after the six heating–cooling cycles of the accelerated stability study (Figure 13A), and after 1 month-storage at 4 °C, 30 °C, and 45 °C (Figure 13B,C). The pH of VDL and VFL phytosome gels was not changed during the accelerated and long-term (1-month) stability studies (Figure 14).

**Figure 15:**
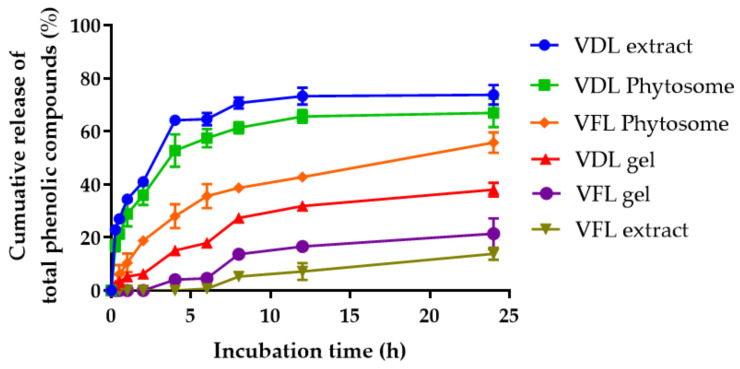
In vitro release curves of VDL and VFL extracts, phytosomes, and shape memory gel loaded with VDL or VFL phytosomes in phosphate buffer saline (PBS) within 24 h.

The sustained release behavior of the total phenolic content from VDL and VFL extracts, phytosomes, and gels was observed (Figure 15). The VDL extract, VDL phytosome, and VDL gel released the total phenolic content up to 73.76 ± 3.73%, 66.98 ± 5.33%, and 38.04 ± 2.59%, respectively, within 24 h. The VFL extract, VFL phytosome, and VFL gel released 13.92 ± 2.26%, 55.78 ± 3.86%, and 21.44 ± 5.81% of total phenolic content, respectively, in 24 h. The results suggested that the VDL extract contained a higher amount of hydrophilic polyphenols compared with the VFL extract, hence releasing the phenolic compounds at a higher rate. The VDL extract entrapped in the phytosomes gradually released total phenolic compounds from the phytosomes. The shape memory gel released phenolic compounds to a lesser extent compared with VDL extract and phytosomes. Interestingly, the VFL phytosome released the phenolic compounds faster than that of the extract and the gel. These results indicated that phytosomes enhanced the water solubility of the VFL extract. Alshahrani et al. showed that phytosomes loaded with *Cuscuta reflexa* extract released 96.3 ± 3.7% of the polyphenol and flavonoids phytoconstituents from phytosomes in 12 h, compared to 49.3 ± 2.5% in the plain extract. Therefore, the phytosomal nanocarriers have the potential to increase the bioavailability of the extract [48]. The release of the phenolic compounds in the gel-loading phytosomes occurred in several steps. The release was initiated by the penetration of the PBS medium into the gel, which created pores and degraded polymers. Then, the phenolic compounds diffused from the gel matrix to the medium, followed by the dissolution of the phenolic compounds in the medium [49].

Shape memory gel is one of the new materials that can fulfill drug delivery and cosmetic application. Polyurethane-62 is a copolymer comprised, in part, of the carbamate (i.e., urethane) linkages that can form hydrogen bonds to yield its high mechanical strength. Combining hydrophobic polyurethane with butylene glycol generates a swellable copolymer network that is robust and durable [50]. Polyurethane-butylene glycol serves as a shape memory polymer. This stimuli-responsive material can memorize its original shape, which occurs during the gelation process when an appropriate stimulus is applied. Biocompatibility and cytotoxicity of the shape memory polymer are crucial concerns for drug delivery. It was suggested that polyurethane could be used for implanted medical devices with shape memory requirements. Peng et al. reported that the polyurethane grafted with poly-lactic acid had biocompatibility comparable to pure PLA [51]. Polyurethane-62 has been widely used in cosmetics, including moisturizers, sunscreens, serums, and water gel lotions. The Australian Industrial Chemicals Introduction Scheme (AICIS) determined that polyurethane-62 was not considered to pose an unreasonable risk to the health of workers and the public [52]. Polyurethan-62 is a high molecular weight polymer. The molecular weight is approximately 100,000 Da, which is not expected to penetrate the skin.

## 3. Conclusions

The phytosomes composed of phosphatidyl choline and cholesterol coated with poloxamer were successfully developed and suitable for the delivery of *N. tabacum* var. Virginia fresh and dry leaf extract to the skin. The phytosomes with different formulars were fabricated and the physical stability was compared. Both phosphatidyl choline and cholesterol played an important role in the physical stability of the phytosomes but did not affect the encapsulation efficiency of the phenolic compounds. VDL and VDL phytosomes had higher phenolic and flavonoid contents and displayed stronger DPPH and ABTS radical scavenging effects, suggesting that phenolic compounds are the main factor in their antioxidant activity. VDL and VFL phytosomes reduced the intracellular ROS and inhibited the NO production induced by LPS. The viscosity and pH of gels containing VDL and VFL phytosomes were not significantly changed after the six heating–cooling cycles of the accelerated stability study and after 1 month storage at 4 °C, 30 °C, and 45 °C.

## 4. Materials and Methods

### 4.1. Materials

Gallic acid, Griess reagent, DPPH (2,2-diphenyl-1-picrylhydrazyl), TPTZ (2,4,6-Tris(2-pyridyl)-s-triazine, and ABTS (2,2′-azino-bis(3-ethylbenzothiazoline-6-sulfonic acid)) were purchased from Sigma-Aldrich, St. Louis, USA. Absolute ethanol, dimethyl sulfoxide, sodium bicarbonate, sodium nitrate, acetic acid, and sodium hydroxide were purchased from RCI Labscan, Bangkok, Thailand. Iron (III) chloride hexahydrate and 37% hydrochloric acid were purchased from Qrec, New Zealand. The Folin–Ciocalteu phenol reagent, aluminum chloride, sodium acetate trihydrate, ferrous sulfate heptahydrate (99% purity), and potassium persulfate were obtained from Loba Chemie, Mumbai, India. Quercetin (98% purity), epigallocatechin (EGCG) (98% purity), ascorbyl glucoside, phosphatidylcholine, cholesterol, poloxamer 407, and polyurethane-62 were purchased from Chanjao Longevity Co., Ltd., Bangkok, Thailand. Dulbecco’s Modified Eagle’s Medium (DMEM) with high glucose, fetal bovine serum (FBS), penicillin-streptomycin, and trypsin-EDTA were purchased from Gibco (Waltham, MA, USA).

### 4.2. Methods

#### 4.2.1. Preparation of VDL and VFL Phytosomes and Nanoparticles

Tobacco leaf extract (VDL or VFL) in 95% ethanol (0.5 g/mL, 400 µL) was mixed with phosphatidylcholine (30 mg in 1900 µL 95% ethanol). Cholesterol (2 mg) in acetone solution (200 µL) was added to the above solution and mixed thoroughly. Then, the mixture was added dropwise into 15 mL of 0.1% *w*/*v* poloxamer 407 at a rate of 1 mL/h with a stirring speed of 700 rpm. The obtained phytosome was named “VDL phytosome” or “VFL phytosome” [53]. “VDL phytosomes (*w*/*o* cholesterol)” and “VFL phytosomes (*w*/*o* cholesterol)” were prepared by the following method, where cholesterol was not included. Tobacco leaf extract (VDL or VFL) in 95% ethanol (0.5 g/mL, 400 µL) was mixed with phosphatidylcholine (30 mg in 1900 µL 95% ethanol). The mixture was added dropwise into 15 mL of 0.1% *w*/*v* poloxamer 407 at a rate of 1 mL/h with a stirring speed of 700 rpm. To prepare “VDL phytosome (*w*/*o* poloxamer)” and “VFL phytosome (*w*/*o* poloxamer)”, mixtures of VDL or VFL (0.5 g/mL, 400 µL) with phosphatidylcholine (30 mg in 1900 µL), 95% ethanol, and cholesterol (2 mg in 200 µL acetone) were infused into 15 mL of de-ionized water at a rate of 1 mL/h with a stirring speed of 700 rpm.

“VDL nanoparticles” and “VFL nanoparticles” were prepared by the infusion of VDL or VFL extracts (0.5 g/mL, 400 µL) into 0.1% poloxamer 407 solution at a rate of 1 mL/h with a stirring speed of 700 rpm [54,55,56]. The obtained VDL and VFL phytosomes and nanoparticles were washed three times with de-ionized water and characterized.

#### 4.2.2. Characterization and Stability Study of VDL and VFL Phytosomes and VDL and VFL Nanoparticles

The freshly prepared VDL and VFL phytosomes or nanoparticles were measured for the size, PDI, and zeta potential values using the Zetasizer (Malvern Instruments, Worcestershire, UK). The colloidal stability of VDL and VFL phytosomes or nanoparticles was studied by storing phytosomes or nanoparticles at 4 °C, 30 °C, and 45 °C for 0.5, 1, 2, and 3 months. At the end of the incubation time, the particle size, PDI, and zeta potential of VDL and VFL phytosomes and nanoparticles were analyzed.

#### 4.2.3. Quantitative Analysis of Total Phenolic Compounds in VDL and VFL Phytosomes

VDL and VFL extracts and phytosomes and gallic acid standard solution (3.9–125 µg/mL) were placed in a 96-well plate (50 µL/well). A Folin–Ciocalteau reagent (10% *v*/*v*, 100 µL) was added to the wells, mixed well, and incubated for 4 min at room temperature. Then, the sodium carbonate solution (10% *w*/*v*, 50 µL) was added to the mixture and incubated in the dark for 60 min at room temperature [57]. The absorbance was measured at a wavelength of 765 nm using a UV-Vis spectrophotometer microplate reader. The total phenolic contents in phytosomes were calculated by constructing a standard curve between the absorbance and the concentration of the gallic acid standard solution. The total phenolic content was expressed as the gallic acid equivalent (GAE).

#### 4.2.4. Quantitative Analysis of Total Flavonoid Content in VDL and VFL Phytosomes

The VDL and VFL extracts and phytosomes, quercetin standard solution (7.8–500 µg/mL), and EGCG standard solution (7.8–1000 µg/mL) were placed in a 96-well plate (50 µL/well). Sodium nitrate (5% *w*/*v*, 30 µL) was added to the wells, mixed well, and incubated for 5 min at room temperature. Then, the aluminum chloride solution (2% *w*/*v*, 50 µL) was added to the mixture and incubated for 6 min at room temperature [57]. Sodium hydroxide (1 N, 50 µL) was added to the wells and incubated for another 10 min at room temperature. The absorbance was measured at a wavelength of 510 nm using a UV-Vis spectrophotometer microplate reader. The total flavonoid contents in phytosomes were calculated by constructing a standard curve between the absorbance and the concentration of quercetin or the EGCG standard solution. The total phenolic content was expressed as the quercetin equivalent or EGCG equivalent.

#### 4.2.5. Chemical Stability Study of VDL and VFL Phytosomes

The chemical stability of VDL and VFL phytosomes stored at 4 °C, 30 °C, and 45 °C for 3 months was investigated by a quantitative analysis of total phenolic and total flavonoid contents in VDL and VFL phytosomes.

#### 4.2.6. DPPH Free Radical Scavenging Assay of VDL and VFL Phytosomes

The VDL and VFL extracts (3.9–20,000 µg/mL), VDL and VFL phytosomes (26.0–13,333 µg/mL), gallic acid solution (3.9–2000 µg/mL), quercetin solution (3.9–2000 µg/mL), EGCG solution (3.9–2000 µg/mL), and ascorbyl glucoside solution (3.9–2000 µg/mL) were added to 96-well plates (100 μL/well). The DPPH solution (0.1 mM) was added to the samples (100 µL/well). The mixtures were incubated in the dark at room temperature for 30 min [56]. Then, the absorbance at a wavelength of 517 nm was measured. The DPPH radical scavenging activity of samples was calculated by the following equation.

$$DPPH\;radical\;scavening\;activity\;\left(\%\right)=\frac{1\;–\;A_{Sample}}{A_{Control}}\times 100$$

#### 4.2.7. ABTS Free Radical Scavenging Assay of VDL and VFL Phytosomes

The VDL and VFL extracts (3.9–20,000 µg/mL), VDL and VFL phytosomes (26.0–13,333 µg/mL), gallic acid solution (3.9–2000 µg/mL), quercetin solution (3.9–2000 µg/mL), EGCG solution (3.9–2000 µg/mL), and ascorbyl glucoside solution (3.9–2000 µg/mL) were added to 96-well plates (20 μL/well). The ABTS•+ solution (0.1 mM) was added to the samples and standard solutions (180 µL/well) [56]. The mixtures were incubated in the dark at room temperature for 15 min. Then, the absorbance at a wavelength of 734 nm was measured. The ABTS•+ radical scavenging activity of the samples was calculated by the following equation.

$$ABTS\;radical\;scavening\;activity\;\left(\%\right)=\frac{1\;–\;A_{Sample}}{A_{Control}}\times 100$$

#### 4.2.8. Ferric Reducing Antioxidant Power Assay of VDL and VFL Phytosomes

The VDL and VFL extracts (3.9–20,000 µg/mL), VDL and VFL phytosomes (26.0–13,333 µg/mL), gallic acid solution (3.9–2000 µg/mL), quercetin solution (3.9–2000 µg/mL), EGCG solution (3.9–2000 µg/mL), and ascorbyl glucoside solution (3.9–2000 µg/mL) were added to 96-well plates (20 μL/well). The FRAP reagent, consisting of the acetate buffer (300 mM, pH 3.6), ferric chloride (20 mM), and TPTZ (10 mM) mixture at a ratio of 10:1:1, was added to the sample solutions (180 µL/well). The mixture of samples and FRAP reagent was then incubated at 37 °C for 30 min before reading the absorbance at a wavelength of 595 nm (Spectramax M3, Molecular Devices, San Jose, CA, USA) [58]. The FRAP values were calculated from the linear equation of a standard curve plotted between the concentration of the ferrous sulfate standard solution (9.8–5000 µM) and the absorbance value at 595 nm.

#### 4.2.9. HPLC Analysis of Bioactive Compounds in VDL and VFL Extracts

HPLC separation was achieved on the HPLC (Agilent, Santa Clara, CA, USA) equipped with the 1260 Infinity II quaternary pump, 1260 Infinity II autosampler, 1260 Infinity II multi-column thermostat, and 1260 Infinity II PDA detector. The separation was completed in an ACE 5 C18-AR column (4.6 × 250 mm i.d., 4.6 mm) with a C_18_ guard column. The mobile phases were (B) acetonitrile and (C) 0.085% phosphoric acid in water using the following gradient elution: 10% B in C to 30% B in C for 15 min; 10% B for 5 min before each analysis, and the flow rate was set at 0.7 mL/min with the controlled temperature at 25 °C. The UV detector was set at the wavelength of 326 nm for the chlorogenic acid analysis and 356 nm for the rutin analysis, and the injection volume was 10 µL for every sample and reference standard [38,39].

#### 4.2.10. Cell Culture

HaCaT and RAW264.7 cell lines were obtained from Dr. Kanokwan Kiettisin and Dr. Natthachai Duangnin, respectively. Cells were cultured in Dulbecco’s Modified Eagle’s Medium (DMEM) with high glucose and supplemented with 10% FBS and 1% penicillin-streptomycin. Cells were incubated in 5% CO_2_ at 37 °C for growing. Cells were subcultured by incubating with 0.25% trypsin-EDTA every three days.

#### 4.2.11. Cytotoxicity Assay of VDL and VFL Phytosomes against HaCaT Keratinocyte Cells

In 96-well plates, HaCaT cells were seeded in a culture medium at 8 × 10^3^ cells/well. Prior to an MTT assay, cells were allowed to adhere for 24 h. VDL and VFL extracts and VDL and VFL phytosomes were added to the cells and were incubated in 5% CO_2_ at 37 °C for 24 h. After treatment for 24, 48, and 72 h, an MTT solution in the culture medium (0.5 mg/mL) was added to each well and incubated for 2 h at 37 °C [58]. The formazan products resulting from the viable cells’ metabolism were dissolved in DMSO (100 µL/well). The absorbances were measured at 550 nm. The IC_50_ values were calculated using GraphPad Prism v.7.0 (La Jolla, CA, USA). Cell viability was calculated using the following equation.

$$Cell\;viability\;\left(\%\right)=\frac{A550\;sample}{A550\;control}\times 100\%$$

#### 4.2.12. Flow Cytometry Analysis of Intracellular Reactive Oxygen Species (ROS)

HaCaT cells were trypsinized and added into the microcentrifuge tubes (1 × 10^6^ cells/mL). Cells were co-incubated for 2 h at 37 °C with 20 mM hydrogen peroxide and VDL or VFL phytosomes at different concentrations. Cells were washed three times with PBS, pH 7.4, followed by incubation with 10 μM DCFH-DA at 37 °C for 30 min [59]. The fluorescent intensity was detected by flow cytometry (Accuri, BD, Franklin Lakes, NJ, USA). For each sample, 10,000 events were recorded.

#### 4.2.13. In Vitro Anti-Inflammatory Assay of VDL and VFL Phytosomes

The viability of RAW264.7 cells after the 24 h incubation with VDL and VFL phytosomes (7.8–1000 µg/mL) was tested by the MTT assay. The anti-inflammatory effect of VDL and VFL phytosomes was determined from nitric oxide secretion against macrophage cells. RAW 264.7 cells (1 × 10^4^ cells/well) were incubated with LPS (50 ng/mL) in the presence or absence of VDL and VFL phytosomes (15.6–500 µg/mL) for 18 h at 5% CO_2_, 37 °C. After incubation, an equal medium volume was mixed with the Griess reagent, consisting of 20 mg/mL sulfanilamide and 1 mg/mL N-(1-naphthylethylenediamine in 5% phosphoric acid at a 1:1 ratio [60,61]. The absorbance of the cell supernatant was recorded at 540 nm to quantify the nitrite levels using a UV-Vis spectrophotometer microplate reader (Spectramax M3, Molecular Devices, San Jose, CA, USA) equipped with SoftMax^®^ Pro 7 software. The amount of nitrite was calculated from the sodium nitrite standard curve. The treated cells were then tested for cell viability using an MTT assay. The medium was replaced with 0.5 mg/mL MTT reagent (100 µL/well) and incubated for 2 h. The formazan product was measured at 550 nm using a UV-Vis spectrophotometer microplate reader (Spectramax M3, Molecular Devices, San Jose, CA, USA).

#### 4.2.14. Formulation of Shape Memory Gel Containing VDL and VFL Phytosomes

The shape memory gel was formulated by mixing polyurethane-62-butlyelene glycol (5%) in de-ionized water (89.4%) at 50 °C with a stirring rate of 1200 rpm for 1 h. Then, glycerin (5%), phenoxyethanol (0.5%), and VDL and VFL phytosomes (0.1 %) were added to the gel.

#### 4.2.15. Characterization and Stability Study of Shape Memory Gel

The pH and rheology of the shape memory gel containing VFL and VFL phytosomes were measured using a pH meter and rheometer (AMETEK Brookfield, Middleboro, MA, USA) equipped with a plate and plate geometry, respectively [62]. The physical stability of the products was investigated by measuring the pH and rheology of the gel after six heating/cooling cycles and after storage at 4 °C, 30 °C, and 45 °C for 1 month.

#### 4.2.16. Release Study of VDL and VFL Extracts, Phytosomes, and Shape Memory Gel

The release profile of the VDL and VFL extracts, VDL and VFL phytosomes, and shape memory gels containing VDL and VFL phytosomes were evaluated by the method described by Liu et al. [63]. Phytosomes containing the same concentration of VDL and VFL extracts (13.33 mg/mL, 200 µL) and gels containing 0.1% VDL and VFL phytosomes were put in the upper chamber of Transwell^®^ (Corning, Glendale, AZ, USA), where the donor chamber contained the PBS buffer (1000 µL). The total phenolic compounds released from extracts, phytosomes, and gels were collected at predetermined time intervals, i.e., 15 min, 30 min, 1, 2, 4, 6, 8, 12, and 24 h. The total phenolic contents were measured by the Folin–Ciocalteau method.

#### 4.2.17. Statistical Analysis

The data were statistically analyzed using a one-way ANOVA, followed by the Newman–Keuls method as a post hoc test to determine the significance of differences (GraphPad Prism 7.02, La Jolla, CA, USA). In all cases, *p* < 0.05, *p* < 0.01, *p* < 0.001, and *p* < 0.0001 were deemed statistically significant. Data were presented as the mean ± SD of the % cell viability (*n* = 3).

## Figures and Tables

**Table 1:** Pearson correlation coefficients of total phenolic content and total flavonoid content, and antioxidant activities of VDL, VFL, VDL phytosomes, and VFL phytosomes measured by DPPH, ABTS, and FRAP assays.

Antioxidant Assay	Total Phenolic Content (Gallic Acid Equivalent)				Total Flavonoid Content (Quercetin Equivalent)			
	VDL	VDL Phytosome	VFL	VFL Phytosome	VDL	VDL Phytosome	VFL	VFL Phytosome
DPPH assay	0.9090 ****	0.9823 ****	0.9557 ****	0.9773 ****	0.7717 *	0.9733 ****	0.9166 ****	0.9682 ****
ABTS assay	0.9259 ****	0.9422 ****	0.9862 ****	0.9830 ****	0.8013 *	0.9094 **	0.9663 ****	0.9015 **
FRAP assay	0.9907 ****	0.9932 ****	0.9901 ****	0.9697 ****	0.9192 **	0.9876 ****	0.9995 ****	0.9982 ****

* indicated *p* < 0.05, ** indicated *p* < 0.01, and **** indicated *p* < 0.0001.

## Supplementary Materials

The following are available online at https://www.mdpi.com/article/10.3390/gels9020078/s1, Figure S1: Calibration curves of gallic acid, quercetin, and epigallocatechin (EGCG); Figure S2: Appearance of shape memory gel containing 0.1% *w*/*w* of VDL phytosomes and VFL phytosomes.
