# Supplementary material for: Antioxidant, Anti-Inflammatory and Attenuating Intracellular Reactive Oxygen Species Activities of Nicotiana tabacum var. Virginia Leaf Extract Phytosomes and Shape Memory Gel Formulation
Source: Gels. 2023 Jan 18;9(2):78. doi: 10.3390/gels9020078 (PMC9956251; doi:10.3390/gels9020078)
Supplement: Supplementary file 1 [file gels-09-00078-s001.zip › gels-2162558-supplementary.pdf]

gels

MDPI

Article

Antioxidant, Anti-Inflammatory and Attenuating Intracellular Reactive Oxygen Species Activities of *Nicotiana tabacum* var. Virginia Leaf Extract Phytosomes and Shape Memory Gel Formulation

Chuda Chittasupho, Kunyakorn Chaobankrang, Araya Sarawungkad, Weerasak Samee, Sudarshan Singh, Kirachuda Hemsuwimon, Siriporn Okonogi, Kantaporn Kheawfu, Kanokwan Kiattisin and Wantida Chaiyana

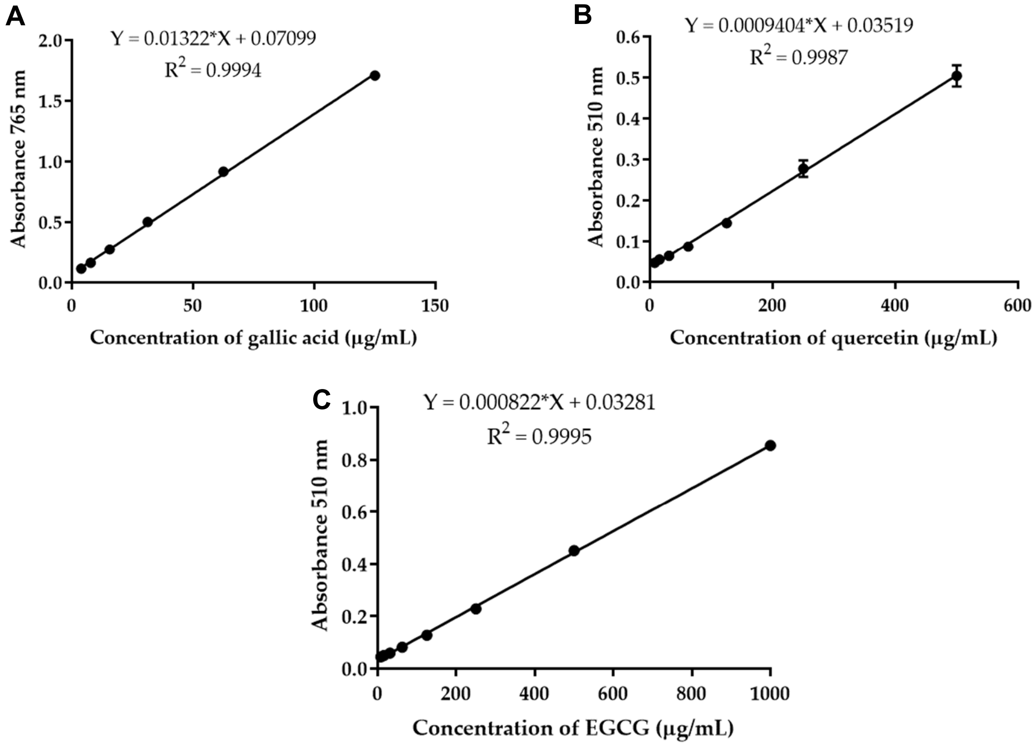

Figure S1. (**A**) Calibration curve of gallic acid (**B**) quercetin and (**C**) epigallocatechin (EGCG).

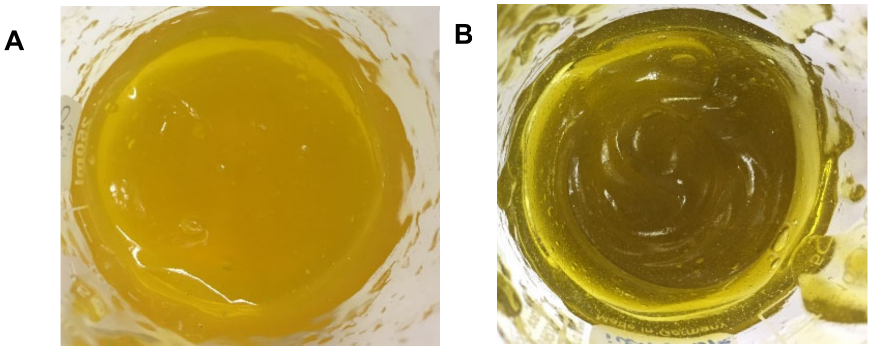

Figure S2. Appearance of shape memory gel containing 0.1% w/w of (**A**) VDL phytosomes and (**B**) VFL phytosomes.
